# Exploring genome wide bisulfite sequencing for DNA methylation analysis in livestock: a technical assessment

**DOI:** 10.3389/fgene.2014.00126

**Published:** 2014-05-13

**Authors:** Rachael Doherty, Christine Couldrey

**Affiliations:** ^1^Animal and Bioscience Research Department, Animal and Grassland Research and Innovation CentreTeagasc, Grange, County Meath, Ireland; ^2^Reproductive Technologies, AgResearch Ruakura Research CentreHamilton, New Zealand

**Keywords:** epigenetics, DNA methylation, sheep, quantification, RRBS, WGBS, fragment size

## Abstract

Recent advances made in “omics” technologies are contributing to a revolution in livestock selection and breeding practices. Epigenetic mechanisms, including DNA methylation are important determinants for the control of gene expression in mammals. DNA methylation research will help our understanding of how environmental factors contribute to phenotypic variation of complex production and health traits. High-throughput sequencing is a vital tool for the comprehensive analysis of DNA methylation, and bisulfite-based strategies coupled with DNA sequencing allows for quantitative, site-specific methylation analysis at the genome level or genome wide. Reduced representation bisulfite sequencing (RRBS) and more recently whole genome bisulfite sequencing (WGBS) have proven to be effective techniques for studying DNA methylation in both humans and mice. Here we report the development of RRBS and WGBS for use in sheep, the first application of this technology in livestock species. Important technical issues associated with these methodologies including fragment size selection and sequence depth are examined and discussed.

## INTRODUCTION

DNA methylation analysis has become an important component of the post-genomic agricultural research era. In order to continue making gains in genetic applications, an understanding of how epigenetic modifications affect gene expression and the resulting phenotype is required. Recent technological advancements, including the application of next generation sequencing strategies, have aided in the progress in the field of epigenetics (Berry et al., 2011; Bai et al., 2012). DNA methylation is difficult to analyze experimentally as it does not alter the DNA sequence and is not maintained during polymerase chain reaction (PCR) cycling because DNA polymerase does not distinguish between methylated and unmethylated cytosines (Kristensen and Hansen, 2009). To detect site specific DNA methylation levels, bisulfite treatment of DNA is still commonly used. Bisulfite treatment converts unmethylated cytosine residues into uracil while methylated cytosines remain unchanged (Frommer et al., 1992). Methylation may then be assessed by restriction enzyme digestion, sequencing, or mass spectrometry. However, the application of this approach at a whole genome level remains costly for organisms with large genomes including mammalian species (Smith et al., 2009). In human and mouse research, the application of reduced representation bisulfite sequencing (RRBS) methods have allowed for genome wide DNA methylation analysis with reduced sequencing requirements, thereby making studies with multiple replicates, group comparisons or cohort studies more achievable and affordable (Boyle et al., 2012). The RRBS methodology, designed by Meissner et al. (2005), Gu et al. (2011) allows for preferential selection and sequencing of CpG-rich regions whilst CpG-poor intergenic regions are under-represented in the library. This results in the sequencing of a subset of DNA fragments from the genome which is likely to contain the majority of regions relevant for DNA methylation analysis without the sequencing of regions that are devoid of CpG sites reducing the cost. In RRBS, the subset of DNA fragments is obtained by digesting genomic DNA with a restriction enzyme (usually MspI, which has a recognition sequence of 5′-C^^^CGG-3′), therefore every fragment produced will contain at least one CpG dinucleotide. Genes, promoters and CpG islands are overrepresented in the fragment subset due to the higher frequency of MspI recognition sites in these CpG-rich regions of the genome. By using or combining different restriction enzymes, CpG coverage across the genome can be altered to include or exclude certain regions of interest such as CpG island shores, which are known to play an important role in various biological processes including cellular differentiation (Doi et al., 2009; Wang et al., 2013).

Since the original development of this technique, systematic assessment of the application of RRBS has been carried out in humans, including examination of genome coverage, mean coverage depth and reproducibility (Wang et al., 2012). Whilst RRBS methodologies have been developed using human and particularly mouse DNA samples, the technique should transfer well to other mammalian species (Smith et al., 2009) and could in theory be applied to animals of agricultural importance including sheep and cattle. *In silico* prediction methods can aid in the design of these studies (Chatterjee et al., 2013) by bioinformatically predicting the number of enzyme cut sites and the distribution of these sites across the genome of interest. *In silico* digestion can also aid in the selection of fragment sizes for sequencing, after the genomic DNA has been cut with the restriction enzymes (Couldrey et al., unpublished data). For vertebrate genomes, it has been indicated that a fraction of DNA fragments between 40 and 220 bp contains enrichment of most promoter sequences and CpG island regions (Meissner et al., 2008; Gu et al., 2010). However, as utilization of epigenomic technologies in livestock species remain under-utilized, application of this technology has yet to be thoroughly explored and verified in practice. This study was undertaken to investigate the application of RRBS in sheep. Some of the issues addressed include expected mapping efficiencies, the best fragment sizes to select for sequencing and importantly the optimum amount of sequencing required to obtain sufficient information, whilst remaining cost effective. In addition, to complement this analysis and as a result of reducing sequencing costs, a comparison between RRBS and the unbiased method of whole genome bisulfite sequencing (WGBS) was carried out. The overall aim of this paper is to address some of the technical issues associated with the application of RRBS technology in livestock species and to aid in the design and implementation of future epigenomic studies.

## MATERIALS AND METHODS

### DNA EXTRACTION, RESTRICTION DIGEST, AND ADAPTOR LIGATION

A *longissimus dorsi* (LD) muscle sample from a wild-type 8 month old Poll Dorset lamb was collected and high quality DNA extracted (Sambrook et al., 1989). RRBS methodology (based on previously published RRBS studies (Cokus et al., 2008; Smith et al., 2009) was used to quantify DNA methylation levels across the genome. MspI restriction enzyme was used to digest 5 μg genomic DNA in 200 μl water with the appropriate reaction buffer at 37°C overnight. The extent of digestion was checked by electrophoresis of 4 μl DNA digestion reaction on a 1% agarose gel and visualized using SyberSafe (Life Technologies, NZ). If a clear smear with a satellite band at approximately 230 bp was observed then the remainder of the digestion was cleaned using DNA Clean and concentrator^TM^-25 columns (Zymo, Irvine, CA, USA), DNA eluted in 36.5 μl H_2_O and this total volume was used for library preparation. The sticky ends produced by MspI digestion were filled with CG nucleotides and Illumina sequencing adapters (Illumina, CA, USA) containing methylated cytosines, instead of standard adaptors contained in Illumina TruSeq library preparation kit, were ligated onto digested DNA following the manufacturer’s protocols (Illumina TruSeq library preparation kit). Ligation reactions were purified using DNA Clean and concentrator^-TM^-5 columns (Zymo, Irvine, CA, USA) and eluted in 18 μl H_2_O. For WGBS analysis, the original DNA sample was sonicated rather than undergoing restriction digestion so that the entire genome was represented in the library. Sonication conditions were as follows: four cycles of pulse for 1 min 30 s followed by a rest of 1 min 45 s on an amplitude of five using a Misonix sonicator ultrasonic processor XL2020 (Farmingdale, NY, USA).

### FRAGMENT SIZE SELECTION

Size selection was performed manually using 15 μl of the purified ligation reation on a 3% nusieve agarose gel (Alphatech, NZ) to obtain inserts without exposing digested DNA to UV light so as not to fragment the DNA further. The lane containing a 50 bp DNA ladder (Life Technologies) was removed from the gel, stained in an Ethidium Bromide solution and visualized under UV light. Ladder bands of 250–350 bp in size were marked with a pipette tip and removed from the UV light. The ladder was then realigned with the remaining gel and the appropriate gel sliver excised to capture insert sizes of 150–250 bp. This process was repeated to capture insert sizes of 50–150 bp and 250–350 bp in order to determine mapping and coverage obtained after sequencing different insert sizes by RRBS. DNA was purified from gel slivers using gel purification columns (Zymo, Irvine, CA, USA) and eluted in 26 μl H_2_O for WGBS, sonicated DNA was size-selected in a similar manner. Insert sizes of 300–400 bp were isolated for library construction.

Efficiency of adaptor ligation and size selection was determined by qualitative PCR using 1 μl gel purified DNA and 15, 20, and 25 PCR cycles and PCR primers supplied in Illumina TruSeq kit. If PCR products were not clearly seen after 15 cycles then ligation efficiency was deemed not sufficient to proceed.

### BISULFITE CONVERSION

Bisulfite conversion of non-methylated cytosines was performed on 20 μl size-selected fragments using an EZ-DNA bisulfite conversion kit (Zymo, Irvine, CA, USA) following the manufacturer’s instructions, except for a modification to bisulfite conversion conditions as recommended by Smith et al., 2009: 99°C for 5 min, 60°C 25 min, 99°C 5 min, 60°C 85 min, 99°C 5 min, 60°C 175 min, 6 × (95°C 5 min, 60°C 90 min). Bisulfite treated DNA was eluted in 24 μl. Small scale test PCR amplification using primers in Illumina TruSeq kit was performed on 1 μl of converted DNA using 15, 20, 25 PCR cycles to determine the minimum amount of amplification to be performed. The remainding 20 μl of bisulfite treated DNA was amplified for 15–20 PCR cycles in four 100 μl reaction volumes. All PCR reactions for RRBS and WGBS were purified using Clean and concentrator^TM^-5 column (Zymo, Irvine, CA, USA), analyzed on a bioanalyzer (Agilent, Santa Clara, CA, USA) and each library was sequenced on one lane of an Illumina HiSeq sequencer using 100 bp paired-end reads (National Centre for Genome Resources, Santa Fe, NM, USA). RRBS was performed in duplicate for one sample to determine the repeatability.

### BIOINFORMATIC ANALYSIS

Quality control of data was undertaken using FastQC software (Babraham Bioinformatics, UK). Quality and adapter trimming for all samples was carried out using Trim Galore software, which was run in -RRBS mode for the RRBS samples. A Phred score of 20 was used as the quality cut-off value as this is the community accepted value and relates to a 1/100 chance of the assigned nucleotide being in correct, this provided a useful balance between using only high quality DNA without discarding too much sequence. To analyze the relationship between sequencing depth and CpG coverage within promoters, genes and CpG islands, sequence data generated from both RRBS and WGBS strategies was sampled at random from the fastq file using a script developed in house. This sampling created smaller datasets originating from the same library and sequence file. Sequences were mapped using paired end mapping to sheep genome assembly OARv3 using Bismark software (Babraham Bioinformatics, UK) which utilizes the Bowtie short read aligner (Langmead et al., 2009). After considerable optimisation, a seed length of 50 bp was chosen and only one mismatch was tolerated. Only sequences in which both ends mapped uniquely with an appropriate insert size in the correct orientation were used for subsequent calculation of DNA methylation levels. Sequencing read counts and levels of methylation were calculated and visualized using Seqmonk software (Babraham Bioinformatics, UK). Analysis of the CpG site coverage across the whole genome, as well as within genes and promoter regions was performed using Seqmonk. The number of CpG sites within these regions that were represented by 1x and ≥10x coverage was identified. Promoter regions were defined as 2 kb upstream of the transcription start site.

### THE RELATIONSHIP BETWEEN DEPTH OF SEQUENCING AND COVERAGE OF CpG SITES

In order to investigate the importance of sequencing depth, the sequence data described above (One lane of each RRBS 150–250 bp insert size and WGBS libraries ~30 GB) was quality and adapter trimmed before being randomly sampled using an in house script, resulting in five sequentially smaller fastq files (25, 18.5, 12, 5, and 2.5 GB) to mimic, *in silico* the number of reads that would be expected, if up to 12 samples were sequenced per lane.

## RESULTS

### QUALITY CONTROL AND MAPPING EFFICIENCIES FOR LIBRARIES PREPARED FROM DIFFERENT DNA FRAGMENT LENGTHS

**FIGURE 1:**
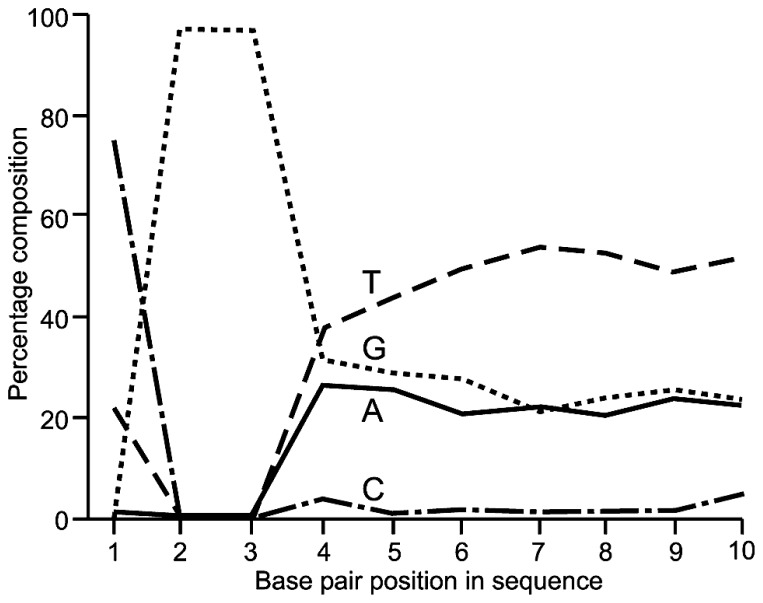
**Base pair composition showing the first 10 bp from read one Illumina HiSeq 100 bp paired end sequencing indicating the expected MspI restriction site at the 5’ end of ~97% of fragments sequenced**.

Quality control analysis using FastQC indicated that for all three fragment sizes analyzed by RRBS, the 100 bp sequences displayed the expected nucleotide composition. On average 97% of read 1 sequences began with CGG or TCC with remaining read 1 sequence being very C poor and T rich rich (an example of the first 10 bp is shown in **Figure 1**). Similarly ~97% of read 2 sequences started with CAA with the remaining sequence being G poor and A rich. Combined non-CpG methylation was for each RRBS and WGBS library was <1% indicating a bisulfite conversion efficiency of >99%.

**FIGURE 2:**
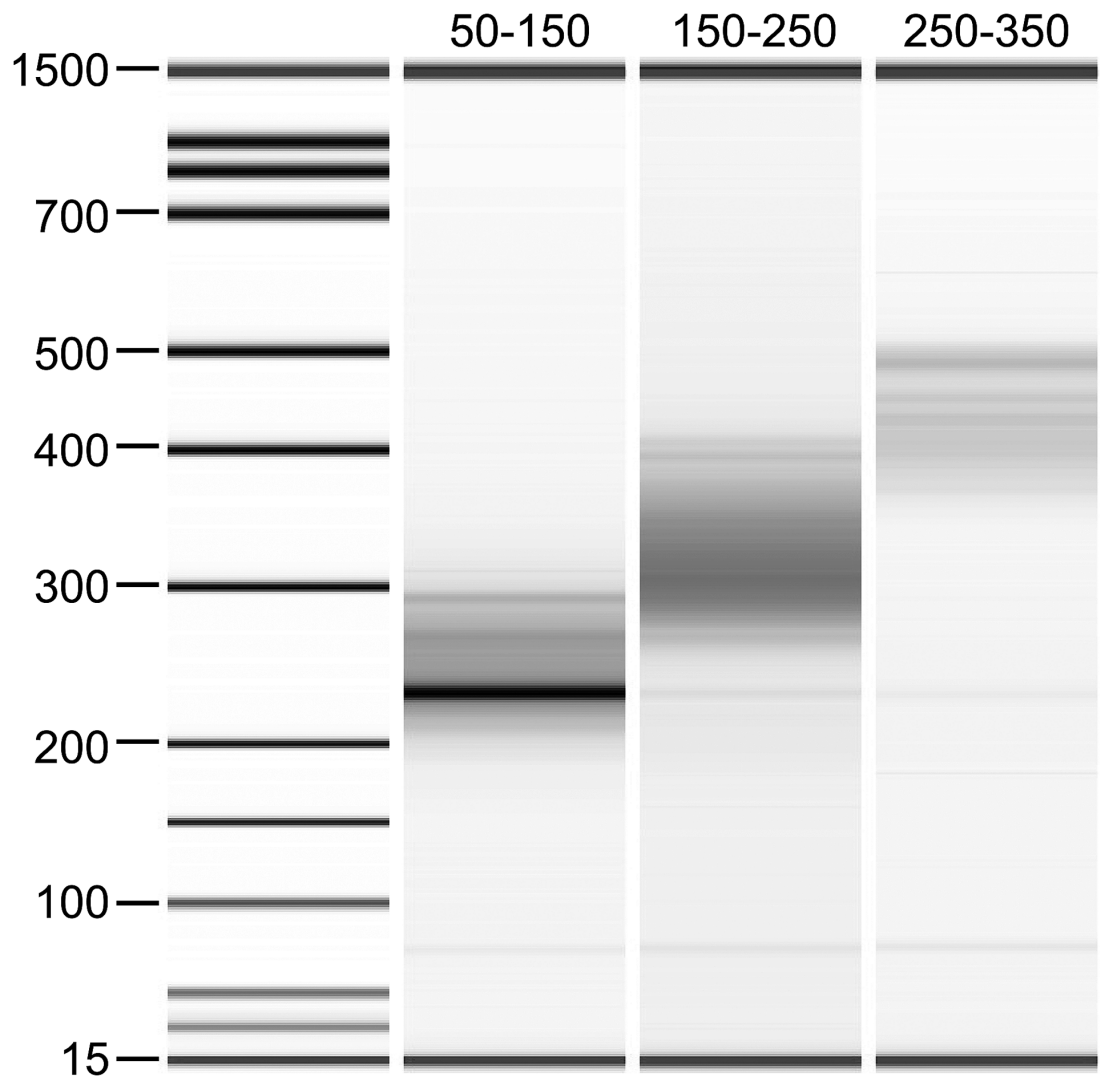
**Bioanalyzer gel image of the three RRBS libraries made with different insert sizes**. Ligated adapters cause the DNA fragments to migrate to a higher molecular weight (approximately 100 bp higher) than the insert sizes selected.

In order to examine the relevance of the size selection process of the DNA fragments on the downstream analysis of DNA methylation in sheep, libraries were made to contain insert sizes of approximately 50–150, 150–250, and 250–350 bp from the same DNA sample (**Figure 2**). Sequence quality and read number were shown to be comparably high for all three libraries using FastQC. Similar numbers of reads were generated for each of these libraries with 109,427,218 reads for the 50–150 bp library, 119,518,539 reads for the 150–250 bp library and 118,713,292 reads for the 250–350 bp library obtained. However, when data were mapped to the sheep reference genome (OARv3.1), a large difference in mapping efficiency was observed for the smallest insert library (50–150 bp) with only 38.3% efficiency compared with the other two libraries, which were 61.4 and 61.7% for insert sizes of 150–250 and 250–350 bp, respectively (**Table 1**).

**Table 1:** Mapping efficiencies and CpG coverage of libraries created with different insert sizes: 50–150, 150–250, and 250–350 bp.

Insert size (bp)	% Mapping	Total no. CpGs	CpGs with ≥ 10x coverage
50–150	38.3	2,094,731	1,067,789
150–250	61.4	3,264,576	1,711,904
250–350	61.7	2,104,633	1,346,714

*For reference, the whole genome is estimated to contain ~28,000,000 CpG sites.*

### CpG COVERAGE AS A RESULT OF DIFFERENT INSERT SIZE SELECTION

The total number of CpG sites sequenced for each insert size was compared. Whilst the 150–250 and 250–350 bp inserts had comparable mapping efficiencies, the amount of informative data generated for CpG methylation analysis was notably different, with the 150–250 bp insert library resulting in the largest number of sequenced CpG sites (**Table 1**). Focusing on CpG sites which were covered by at least 10 reads (minimum number of reads required for accurate determination of DNA methylation if individual CpG site analysis is undertaken), the 150–250 bp insert library resulted in 1,711,904 unique CpGs compared to 1,346,714 unique CpGs originating from the 250–350 bp insert library (**Table 1**).

### GENE AND PROMOTER COVERAGE AS A RESULT OF DIFFERENT INSERT SIZE SELECTION

**FIGURE 3:**
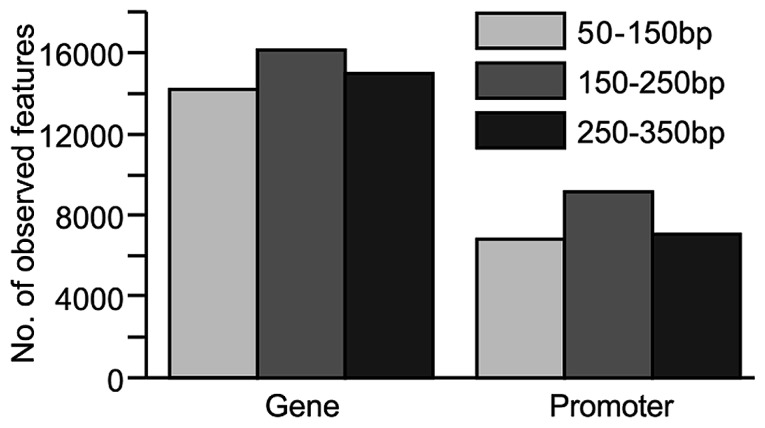
**Comparison of coverage at genomic features (genes and promoter regions) from sequence data generated from libraries constructed from various fragment sizes (50–150, 150–250, and 250–350 bp)**. For inclusion, the genomic feature had to contain at least three CpG sites with ≥10x coverage. Promoter regions were defined as 2 kb upstream of the transcription start site.

The number of CpG sites found within gene bodies (as annotated in OARv3.1) and promoter regions (defined as 2 kb upstream of the transcription start site) was also assessed, as these regions are likely to contain DNA methylation patterns important for gene regulation. For genes or promoter regions to be included in this analysis, they were required to contain at least three CpG sites. In addition, these CpG sites were required to have at least 10x coverage. The 150–250 bp insert library, which provided the greatest CpG coverage across the genome, also appeared to provide the greatest CpG coverage within gene and promoter regions (**Figure 3**).

### MAPPING EFFICIENCIES, CpG COVERAGE, AND DNA METHYLATION LEVELS FOR LIBRARIES PREPARED FOR RRBS VERSUS WGBS

A direct comparison was carried out to examine the data obtained for DNA methylation studies generated from the RRBS library with an insert size of 150–250 bp versus WGBS. Both the RRBS and WGBS libraries were sequenced on one lane of an Illumina HiSeq 2000 sequencer with sequence quality and read number being comparably high for both libraries. RRBS data had a higher mapping efficiency than the WGBS data with a percentage mapping efficiency of 52.3% compared with 42.2% for the WGBS dataset (**Table 2**).

**Table 2:** Mapping efficiencies, CpG coverage and average genome-wide methylation levels resulting from reduced representation bisulfite sequencing (RRBS) and whole genome bisulfite sequencing (WGBS) libraries.

Method	% Mapping	Sequence reads	Total no. CpGs	CpGs with ≥ 10x coverage	% Methylated CpGs
RRBS	52.3	119,518,539	2,599,828	1,765,542	53.5
WGBS	42.2	131,960,496	9,719,824	2,840,025	64.9

*Both library types were sequenced over one lane on an Illumina HiSeq 2000.*

**FIGURE 4:**
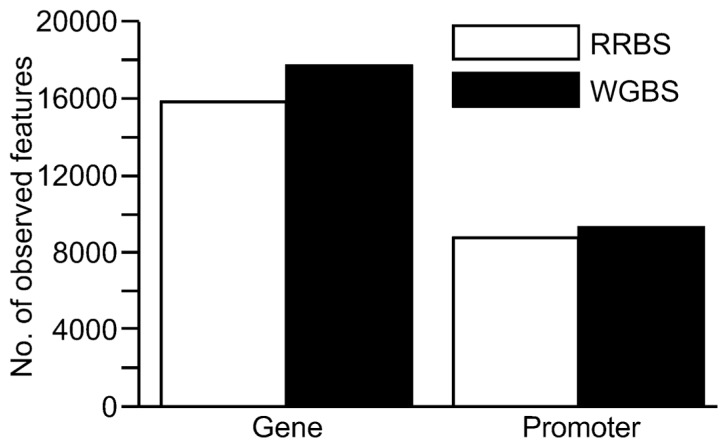
**Comparison of coverage at genomic features (genes and promoter regions) from sequence data generated from a reduced representation bisulfite sequencing (RRBS) library versus a whole genome bisulfite sequencing (WGBS) library**. For inclusion, the genomic feature had to contain at least three CpG sites with ≥10x coverage. Promoter regions were defined as 2 kb upstream of the transcription start site.

The total number of CpG sites sequenced was substantially higher using the WGBS method with a total of 9,719,824 CpG sites covered compared to 2,599,828 for RRBS. However, the number of sequenced CpG sites for the two methods were more similar when a minimum coverage threshold of 10x was applied to the analysis. Introducing the ≥10x cut-off for inclusion of CpG sites resulted in 2,840,025 CpGs for WGBS versus 1,765,542 CpGs for RRBS (**Table 2**). When the coverage of gene and promoter regions was examined, results suggested that one lane of WGBS sequencing again resulted in the inclusion of a greater number of these genomic features than RRBS (**Figure 4**). Across the genome, the average levels of methylation in the samples were found to be 53.5% for the RRBS library and 64.9% for the unbiased WGBS library.

### THE RELATIONSHIP BETWEEN DEPTH OF SEQUENCING AND COVERAGE OF CpG SITES

An important parameter in the design of studies involving bisulfite sequencing methods is the sequencing depth. The impact of sequencing depth on the total number of CpG sites across the whole genome, as well as the local number of CpG sites at promoters and gene bodies were examined. Total CpG coverage as well as the number of CpGs with a coverage depth of at least 10x were analyzed for all files of sampled data. **Table 3** illustrates the observed numbers of CpG sites at 1x and ≥10x coverage for the reduced data sets for what would be expected if up to 12 samples were sequenced in a single lane on an Illumina HiSeq 2000.

**FIGURE 5:**
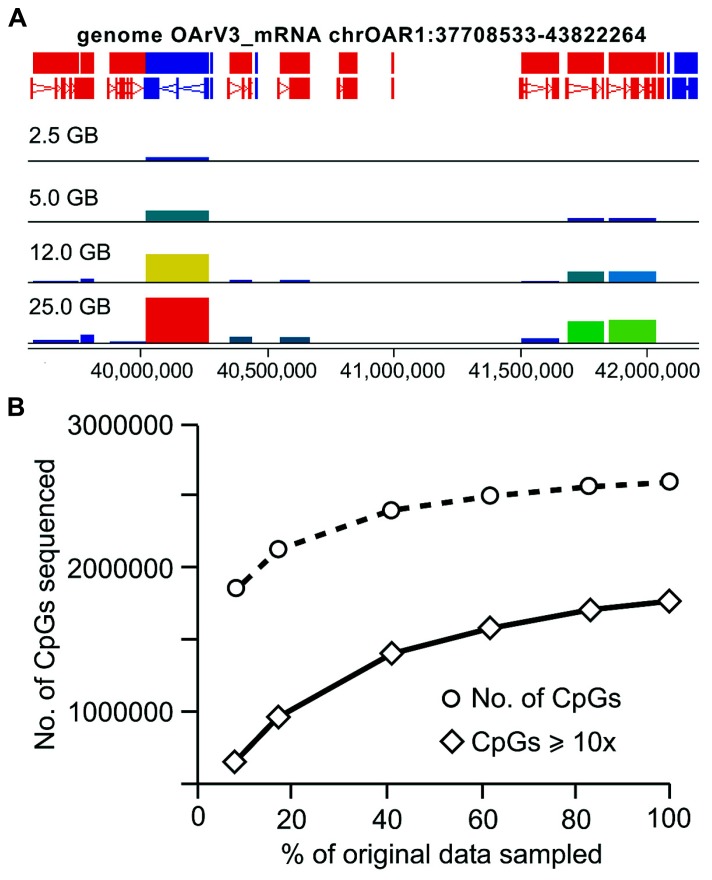
**CpG coverage generated from RRBS when smaller amounts of data are available for analysis**. RRBS data were randomly sampled from the original fastq file to create smaller data sets, **(A)** Seqmonk screen shot illustrating CpG site coverage across selected genes on chromosome 1, bar height represents a count of CpG coverage, illustrating inclusions of increasing number of genes analyzed as the number of sequences included increases; **(B)** The number of CpGs covered in these sequentially smaller datasets was identified, in addition to the number of CpGs with at least 10x coverage.

For RRBS, the total number of annotated genes analyzed and CpG coverage across the genome is proportional to the amount of sequence analyzed (**Figure 5**). When the total amount of sequence drops below 15 GB, CpG coverage is more rapidly reduced than when greater than 15GB of sequence is analyzed. An examination of the coverage of CpG sites with ≥10x coverage depth produced a similar trend, with a more rapid decline in CpG coverage below 15 GB of data (**Figure 5B**). When analyzing only 5 GB of the original 30 GB data set (the equivalent of sequencing six samples/lane as is often offered commercially), 82% of the total number of CpGs are retained. When looking only at the CpGs covered with at least 10x depth, only 54.4% of the original number remained (**Table 3**).

**FIGURE 6:**
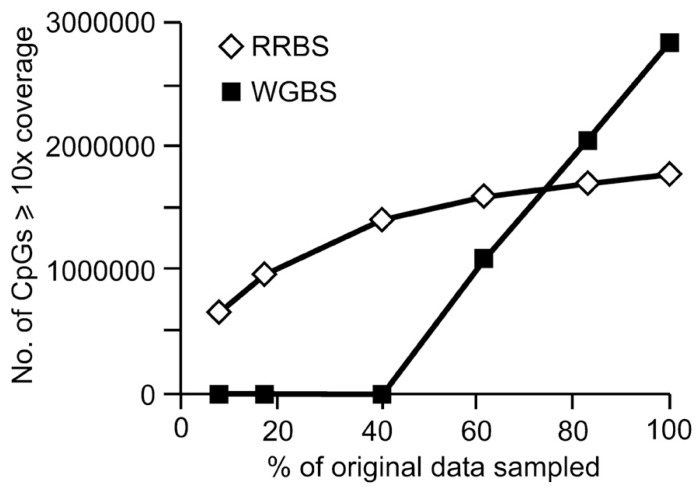
**CpGs with at least 10x coverage in data sets generated from RRBS versus WGBS**.

The coverage trend was markedly different for the WGBS dataset. Whilst the original sized WGBS sequence file yielded more CpG coverage at both 1x and ≥10x coverage depth than the full-sized RRBS dataset, this was not the case when smaller amounts of sequence data were compared. When less data were analyzed, CpG coverage for WGBS rapidly fell (**Figure 6**). When 5 GB of data were analyzed, CpG site coverage was inadequate for a genome wide analysis as only 8059 CpG sites had sufficient coverage to be interrogated. This 5 GB of data, or the equivalent of six samples sequenced in a single lane, is also summarized in **Table 3** showing that when looking only at CpGs with at least 10x depth of coverage, only 0.1% of the original CpGs from the original dataset remained (**Table 3**).

**Table 3:** CpG coverage for reduced representation bisulfite sequencing (RRBS) and whole genome bisulfite sequencing (WGBS) when reduced amounts of sequence data are available.

>Amount of sequencing (GB)	RRBS (total no. CpGs)	Sites covered relative to 30 GB (%)	RRBS (≥10X CpGs)	Sites covered relative to 30 GB (%)	WGBS (total no. CpGs)	Sites covered relative to 30 GB (%)	WGBS (≥10X CpGs)	Sites covered relative to 30 GB (%)
30	2,599,828	100	1,765,542	100	9,719,824	100	2,840,025	100
25	2,566,252	98.7	1,697,946	96.2	9,545,383	98.2	2,037,927	71.8
18.5	2,504,492	96.3	1,584,743	89.8	9,207,310	94.7	1,083,324	38.1
12	2,399,624	92.3	1,400,619	79.3	6,315,859	65.0	8,059	0.3
5	2,128,539	81.9	961,329	54.4	5,851,814	60.2	4,074	0.1
2.5	1,853,981	71.3	645,386	36.6	4,101,353	42.2	1,065	0.0

*Large sequence files (30 GB) obtained from sequencing RRBS and WGBS libraries over one lane of an Illumina HiSeq were randomly sampled resulting in sequentially smaller sequence files for comparison.*

## DISCUSSION

DNA methylation analysis represents a new frontier for animal bioscience research. By mapping the DNA methylome, researchers can examine an epigenetic mechanism responsible for controlling gene expression and determining the fates of developing cells. Bisulfite conversion allows for single nucleotide resolution of absolute methylation levels at CpGs (Stevens et al., 2013) making both RRBS and WGBS attractive choices over other antibody based approaches such as methylated DNA immunoprecipitation sequencing (MeDIP-seq) and methyl-CpG binding domain protein enriched genome sequencing (MBD-seq). RRBS and WGBS analysis relies heavily on next generation sequencing and associated library preparation methods, the unique technical challenges of these techniques in human samples have previously been described (Chatterjee et al., 2012; Wang et al., 2012). This study, using a sheep muscle sample, has dealt with some of the important issues regarding fragment size selection, coverage, depth, and a comparison of sequencing approaches by directly comparing the data from both RRBS and WGBS pipelines, for samples of ovine origin.

When considering multiple factors including mapping efficiency and CpG coverage, size selection of a RRBS insert size of 150–250 bp appears to provide the best dataset for downstream analysis when one lane of Illumina HiSeq 2000 sequence is analyzed. Both the total number of CpG sites and the number of those found within genes and promoter regions were the highest from the library produced from this insert size. The number of CpGs found to have at least 10x coverage was substantially higher for this insert size with an additional 600,000 CpG sites compared to the 50–150 bp inserts and this appears to be reaching the plateau of all captured CpGs being sequenced. The 150–250 bp insert size was also identified as having a satisfactory mapping efficiency of 61.4%. In general terms, mapping efficiencies for RRBS are lower for samples derived from livestock species than those from human and mouse. The reasons for this may be due to a less complete and/or accurate assembly reference genome for sheep and cattle compared to human. Also, the presence of large repeat regions in the genomes of these animals may mean that there are fewer uniquely mapped reads. The need for unique mapping automatically rules out all repetitive RRBS and WGBS products from analysis, leading to a reduction in mapping efficiency. Therefore, the presence of these repeat regions in sheep is likely to be a key factor in the different mapping efficiencies observed for the different insert sizes. Based on the bioanalyzer gel image of the RRBS library generated with smallest insert size (50–150 bp) amplification of a repetitive region is obvious. This repetitive region will be a large contributing factor to the low mapping efficiency observed for this fragment.

A direct comparison of RRBS and WGBS was carried out to assess the value of both approaches. As with insert size comparison, this work also compared sequence data produced from one lane on an Illumina HiSeq 2000, equating to around 30 GB of data. The RRBS dataset had a higher mapping efficiency than WGBS, but a lower average methylation level across the genome. Differences in mapping efficiencies were expected as RRBS datasets are designed to cover a higher proportion of promoters and genes, whereas, the unbiased nature of WGBS means that many more reads originate from regions of poorly assembled non-coding DNA, which can contain large stretches of repeat regions. Differences in genome wide average DNA methylation between the two methods of library construction can also be partly explained by the biasing of RRBS libraries to contain promoter regions. Promoter regions often contain CpG islands, stretches of high CG content known to be largely devoid of DNA methylation (Bird, 2002). RRBS libraries are therefore expected to display lower methylation on average across the genome than unbiased libraries. However, the average methylation of 64.9% calculated from the WGBS library is still lower than the traditionally reported 80% genome wide DNA methylation level (Ehrlich et al., 1982). A possible explanation for this discrepancy is the requirement for unique mapping with both RRBS and WGBS technologies. Repeat regions are generally highly methylated (Bird, 2002) and their exclusion would therefore reduce average methylation calculated across the genome even though they are included for sequencing in WGBS.

Although CpG enrichment occurs in RRBS through using MspI restriction enzyme to ensure that each insert contains at least one CpG site, comparison of an equivalent number of RRBS and WGBS sequence reads identified that coverage of a greater number of CpG sites was achieved using WGBS at both the 1x and ≥10x level. This indicates that at this depth of sequencing, it may be more valuable in terms of relevant data to sequence the whole genome after random sonication, as opposed to using the reduced representation method.

For RRBS, in order to capture sufficient data, it has been recommended by others that a minimum of 3 GB–5 GB of data be acquired for each sample (Li et al., 2010; Wang et al., 2012). Therefore, to quantitatively assess the CpG coverage for smaller datasets generated from sheep, sequence files of sequentially smaller amounts of data were analyzed. For RRBS, the depth and coverage of CpG sites reduced at a steady rate when analyzing greater than of sequence 15 GB (50% illustrated in **Figure 4**), below this the rate of decline was more rapid. For the lowest amount of sequence analyzed (approximately 2.5 GB of data), more than 1.8 million CpG sites were sequenced and 600,000 of these at a depth of at least 10x. In contrast to this, the WGBS dataset provided data for just over 400,000 CpGs at this depth of sequencing and of these only 1065 sites had at least 10x coverage. Therefore, when a full lane of sequencing was available for analysis, WGBS provided information for a greater number of CpG sites than RRBS; however, unless libraries are sequenced to a very high depth, WGBS is an unsuitable approach.

Therefore, WGBS may have a variety of limitations depending on the hypothesis being tested and the study design. For example, if disease-specific epigenetic alterations are being examined, these are typically more subtle than tissue-specific differences or changes related to cellular differentiation (Gu et al., 2010). Therefore, a larger number of biological replicates may be required to detect these differences statistically. In order for this to be financially achievable, lower amounts of sequencing and multiplexing of samples can be employed to sequence multiple samples across a single lane of the sequencer. Alternative approaches are available to measure absolute genome wide methylation levels in humans (Bibikova et al., 2009). These include the array based Infinium methylation assay from Illumina, which has been shown to produce results highly correlated with RRBS (Bock et al., 2010). These array based assays have been the most popular and widely used of the methylomic technologies over recent years (Lowe and Rakyan, 2013). Whilst this human specific technology has been applied to mouse genomic DNA recently with somewhat successful results (Wong et al., 2013), the development of DNA methylation arrays specifically for livestock species is desirable. However, without this, the reliance on RRBS technology remains even greater in animal research. On the basis of our analyzes, RRBS is the method of choice for studying DNA methylation on a large scale in animals of agricultural interest as financial resources are often limiting. RRBS provides reliable estimation of methylation levels at single nucleotide resolution, with sufficient coverage of CpG rich regions including promoters when sequencing depth is limited. Finally, it has previously been shown to generate datasets of a suitable size for genome wide analysis but at a much lower cost than WGBS (Smith et al., 2009), facilitating experiments involving multiple treatments and/or biological replicates. An understanding of DNA methylation, in addition to other epigenetic mechanisms involved in gene regulation, will inevitably aid in our understanding of how epigenetics affects gene expression and ultimately phenotype in animals of agricultural importance. No one solution will be optimal or practical as a blanket solution for measuring DNA methylation in all circumstances, the analysis presented here will provide researchers with information to determine which methodology best suits their needs.

## Conflict of Interest Statement

The authors declare that the research was conducted in the absence of any commercial or financial relationships that could be construed as a potential conflict of interest.
